# Therapeutic Potential of Nutritional Aryl Hydrocarbon Receptor Ligands in Gut-Related Inflammation and Diseases

**DOI:** 10.3390/biomedicines12122912

**Published:** 2024-12-20

**Authors:** Fu-Chen Huang

**Affiliations:** Department of Pediatrics, Kaohsiung Chang Gung Memorial Hospital, Chang Gung University College of Medicine, Kaohsiung 833401, Taiwan; huang817@cgmh.org.tw; Tel.: +886-7-7317123 (ext. 8724); Fax: +886-7-7338009

**Keywords:** aryl hydrocarbon receptor, gut, immunity, diseases

## Abstract

A solid scientific foundation is required to build the concept of personalized nutrition developed to promote health and a vision of disease prevention. Growing evidence indicates that nutrition can modulate the immune system through metabolites, which are either generated via microbiota metabolism or host digestion. The aryl hydrocarbon receptor (AhR) plays a crucial role in regulating immune responses, particularly in the gut, and has emerged as a key modulator of gut-mediated inflammation and related diseases. AhR is a ligand-activated transcription factor that responds to environmental, dietary, and microbial-derived signals, influencing immune balance and maintaining intestinal homeostasis. Nutritional AhR ligands play a significant role in modulating intestinal immunity and the function of mucosal immune cells, thereby exerting clinical effects on colitis and innate immunity. Additionally, they have the capacity to orchestrate autophagy, phagocytic cell function, and intestinal epithelial tight junctions. Therapeutic strategies aimed at enhancing AhR activity, restoring gut integrity, and optimizing immune responses hold promise as avenues for future research and potential treatments for critically ill patients.

## 1. Introduction

Personalized nutrition is a conceptual analog to personalized medicine. The vision of developing personalized nutrition for health promotion and disease prevention requires the construction of a sound scientific basis for this concept. As diet is the most prominent life-long environmental impact on human health and as, with prolonging lifespan and changing lifestyle in developed countries, chronic diseases become more prevalent, nutrigenomics are key scientific platforms to promote health and prevent disease through nutrition that better meets the requirements and constraints of consumer groups with specific health conditions, particular lifestyles, and in certain stages of life. Thomas Edison emphasized a holistic approach to healthcare, suggesting that the future doctor will prioritize educating patients on maintaining a healthy lifestyle, including attention to the human frame and diet, and understand the causes and prevention of disease.

Many naturally occurring compounds derived from fruits and vegetables can act as aryl hydrocarbon receptor (AhR) ligands. For example, the flavonoid quercetin, found in apples, and possibly resveratrol, found in red wine, are among them. This highlights AhR as a significant sensor of environmental cues, capable of initiating transcriptional regulation in response to dietary factors. The AhR is a ligand-activated transcription factor predominantly located in the cytosol, playing a central role in the intestinal mucosa [1].

When binding to an agonist, AhR translocases to the nucleus, where it associates with ARNT (AhR nuclear translocator) to form an active heterodimer. This complex recruits nuclear cofactors to either activate or repress gene expression [2], resembling pathways described for several nuclear receptors. This pathway is like that described for several nuclear receptors. AhR is highly relevant in the gut, where it has an important impact on the function of the immune system [3]. AhR also interacts with other transcription factors during transcription, such as estrogen receptor-α [4], nuclear factor-κB (NF-κB) [5], and vitamin D receptor (VDR) [6]. The interaction between AhR and NF-κB or VDR is bidirectional. AhR agonists have been shown to suppress NF-κB but enhance VDR-mediated gene expression [7].

## 2. Nutritional AhR Ligands

Fruit and vegetables represent an abundant source of naturally occurring AhR ligands (Table 1). For example, the flavonoid quercetin, found in apples, and possibly resveratrol, found in red wine, serve as AhR ligands. Flavonoids are polyphenolic compounds known for their health-promoting properties and are found abundantly in fruits and vegetables. The flavone-based antagonist resveratrol has gained considerable interest due to its apparent beneficial effects on human health. This highlights AhR as an important sensor of environmental cues, capable of initiating transcriptional regulation in response to environmental (dietary) factors. The Brassica genus (broccoli, cauliflower, Brussels sprouts, and cabbages) contains significant amounts of indole-based glucobrassicin. The main class of dietary AhR ligands comprises indoles, encompassing compounds like Indole-3-acetonitrile, Indole-3-carbinole (I3C), 3,3′-diindolylmethane (DIM), and Indole (3,4) bicarbazole. These indoles are primarily abundant in cruciferous vegetables such as broccoli or Brussels sprouts. Additionally, I3C plays a crucial role in the development of gut-associated intraepithelial lymphocytes (IELs) [8] and the preservation of gut epithelial barrier integrity [9]. There’s a suggestion that nutritional AhR ligands may be able to cross the blood–brain barrier following intraperitoneal injection [10]. Microbiota-derived AhR ligands have also been identified in breast milk. Following gavage of pregnant mice with labeled AhR ligands, it was observed that the ligands were transferred from the maternal intestinal microbiota to the milk [11]. I3C is absorbed from the gut and distributed systemically into various well-perfused tissues. While I3C is rapidly cleared from circulation, its condensation products can travel to distant organs and exert longer-lasting effects.

Another source of nutritional AhR ligands is microbiota metabolism, particularly tryptophan (Trp) catabolism. Trp, an essential amino acid obtained exclusively through dietary protein, serves as a precursor to several endogenous metabolites recognized as AhR ligands. Among these metabolites are indole, 2-oxindole, indole-3-acetic acid (I3A), and kynurenic acid (KA), which are derived from Trp metabolism pathways by the gut microbiota. These compounds are considered the primary sources of AhR activation within the gastrointestinal tract [12]. Several studies have highlighted the microbiota as a crucial factor in mediating the degradation of Trp in the large intestine, displaying AhR-dependent anti-inflammatory activities and regulating gut barrier function [12,13]. A notable example is commensal *Lactobacillus reuteri*, which produces the AhR ligand indole-3-aldehyde (IAld). In mice, AhR activation by IAld induces IL-22 production, leading to enhanced restoration of mucosal immune homeostasis [14]. It prevents not only colonization by pathogenic microorganisms like *Candida albicans* [14] but also the worsening of inflammatory disorders affecting the intestinal tract such as inflammatory bowel disease (IBD) and cancer. KA is also identified as a relatively potent ligand for human AhR, and it co-mediates the induction of IL-6 expression along with inflammatory signaling [15].

Short-chain fatty acids (SCFAs), such as butyrate, derived from the fermentation of dietary fibers by the microbiota, can activate AhR signaling. Butyrate activates AhR pathway and AhR-dependent genes in human intestinal epithelial cells (IECs) [16] through a direct AhR activation and probably in complement to its histone deacetylase inhibitory (HDACi) property. These findings suggest butyrate is a potential ligand for AhR which is an original mechanism of gene regulation by SCFAs. Mounting evidence indicates that reduced blood and fecal levels of gut microbiota-derived AhR ligands are associated with many human diseases, such as IBDs, obesity, type 2 diabetes, and high blood pressure [17].

The actions of 1,25-dihydroxyvitamin D3 (1,25D3) are mediated through its interaction with the VDR, a ligand-dependent transcription factor within the nuclear receptor superfamily. Extensive research has highlighted the critical role of 1,25D3 and VDR signaling in regulating diverse biological processes and their involvement in various pathological conditions [18]. VDR is now known to have a broad spectrum of actions as illustrated by a large number of diseases [19]. VDR is in about 30 different tissues and can regulate the expression of more than 1000 genes. Following many fruit- and vegetable-derived ligands binding, AhR can also induce the expression of many AhR-dependent genes. Cooperative regulation of cytochrome P450 enzyme expression by the AhR and VDR has been reported [20]. AhR serves as a molecular target of calcitriol (active form of vitamin D) in human T cells [21]. On the contrary, the suppression of AhR can be mediated through the direct binding of the VDR within the *AHR* gene. In fact, at least 10 potential VDR binding sites have been identified in the *AHR* gene, as indicated by the consensus binding motif. This suggests that VDR may regulate AhR expression through these specific binding interactions [22]. These discoveries imply that the interaction between vitamin D and AhR ligands might play a crucial role in determining the overall outcomes of immune responses. The increasing interest in the epigenetic control of VDR [23] and AhR [24,25] regulation as well as their interaction and its significance for diseases has shown promise. Progress in these areas may lead to the formulation of disease-prevention models based on epigenetic control by individual or associations of food ligands of NRs.

## 3. Nutritional AhR Ligands on Intestinal Immunity

AhR is prominently expressed in epithelial barriers, and research on AhR-deficient (AhR^−/−^) mice has uncovered compromised gut barrier function. This indicates a critical role for AhR in either sustaining or developing healthy gut barriers [26]. Furthermore, the expression of AhR has been shown to protect against intestinal inflammation and maintain barrier function [26]. Experimental intestinal inflammation in mice is ameliorated by many AhR ligands, including I3C [27], flavonoids [28], and a Trp diet [29].

The AhR plays a critical role in maintaining intestinal barrier function by modulating tight-junction integrity and regulating the expression of junction proteins [30,31,32,33]. Upon ligand binding, the AhR translocates to the nucleus, where it induces the expression of target genes. This signaling promotes the production of IL-22 and microbicidal mediators. IL-22, produced in an AhR-dependent manner by immune cells, supports epithelial cell repair, strengthens tight junctions [34], and stimulates the production of antimicrobial peptides [35,36]. By enhancing mucus secretion, promoting epithelial cell differentiation and proliferation, and facilitating antimicrobial protein production, IL-22 is instrumental in preserving gastrointestinal barrier integrity and can influence the microbiome both directly and indirectly [37]. Additionally, exogenous IL-22 treatment in both mice and humans has been shown to alter gut microbiome composition and function, which, in turn, enhances AhR signaling. Increased epithelial damage may be due to the lack of IL22 production. Importantly, AhR activation in IECs is also involved in barrier repair during colitis [38]. The mode of action of both [butyrate and 1,25-dihydroxyvitamin D3] in chronic colitis most likely involves restoring epithelial integrity by regulating tight-junction proteins [7] and rebalancing the homeostasis of the innate and adaptive intestinal immune system. This is achieved by reducing neutrophil and macrophage activity and shifting the Th17/Treg ratio in favor of the Treg subpopulation [39], respectively. Importantly, intestinal microbiota commensals secrete AhR ligands that are crucial for maintaining epithelial integrity and promoting the generation of anti-inflammatory IL-22 by various immune cells.

The interaction between gut microbial and host AhR signaling is bidirectional since modulation of AhR activation can also contribute to alterations in the gut microbial community. Microbial Trp catabolites activate the immune system through binding to the AhR, enhance the intestinal epithelial barrier, exert anti-inflammatory, anti-oxidative effects in systemic circulation, and putatively modulate gut microbial composition to mediate microbe–host interactions as well as stimulate gastro-intestinal motility and secretion of gut hormones [40]. AhR activation by natural AhR ligands (e.g., I3C) has been shown to prevent pathogenic gut microbial dysbiosis by altering gut microbiome composition in murine colitis [41]. Depletion of diet AhR ligands decreased α diversity of gut microbiota, while AhR ligand (e.g., I3C) supplementation restored microbiota composition [42].

The AhR is involved in innate immune responses to microbial invasion of barrier tissues. When AhR is specifically deleted from the intestinal epithelium, it results in a compromised response to *C. rodentium* infection [38,43] and inflammatory damage. This highlights the importance of epithelial AhR expression in maintaining intestinal homeostasis and providing protection. The IL-22-induced secretion of antimicrobial peptides by IECs is able to defense against intestinal infections [11] with *Citrobacter rodentium* [43] or *Clostridium difficile* [44]. On the other hand, AhR-regulated neutrophils and Th17 cells are recruited and activated to generate pro-inflammatory Il-17A and bactericidal reactive oxygen species in epithelial cells [45,46]. The IL-23/IL-22 axis plays a crucial role in innate immunity against *Salmonella* infection, contributing to protection through various mechanisms such as IL-22-regulated expression of antimicrobial peptides and acute-phase proteins, as well as IL-17A-dependent neutrophil recruitment [47].

IL-10 is another potent anti-inflammatory cytokine affected by the AhR. AhR activation induces differentiation of a number of IL-10-producing immune cells [48], such as monocytes, macrophages [49], dendritic cells, NK cells [50], T cells [48,51,52], and neutrophils [53], and promotes IL-10 production [54]. Administration of the AhR agonists induces IL-10 production and suppresses DSS- or trinitrobenzene sulfonic acid (TNBS)-induced colitis in humanized mice [55,56]. Moreover, regulation of the gut immune responses by AhR signaling either increases production of IL-10 to indirectly control the production of tight junction proteins [57] or decreases the detrimental effects of proinflammatory cytokines in regards to disruption of the tight junction proteins [26,31].

Nutritional AhR ligands can modulate the differentiation of immune cells outside of the intestinal mucosa, as shown for monocytes, macrophages and T helper cells. Activation of the AhR promoted monocyte-derived dendritic cells (mo-DCs) differentiation, while impairing differentiation into monocyte-derived macrophages (mo-Macs) [58]. It suggests that AhR acts as a molecular switch for monocyte fate specification in response to micro-environmental factors. The AhR regulates several signaling pathways relevant to intestinal health, including the balance between regulatory T cells (Tregs) and T helper 17 (Th17) cells [39]. Specific effects of AhR ligands on lipopolysaccharide (LPS)-induced genes in bone-marrow-derived macrophages have been observed [59]. AhR ligands exhibit differential effects on cytokine expression in bone-marrow-derived macrophages (BMMs). The compounds 2,3,7,8-tetrachlorodibenzo-p-dioxin (TCDD) and 6-formylindolo[3,2-b]carbazole (FICZ) were shown to induce CCL20 expression, whereas I3C suppressed its expression in lipopolysaccharide (LPS)-activated wild-type BMMs [60]. Similarly, TCDD and FICZ enhanced the LPS-induced expression of IL-6 and IL-10, while I3C significantly inhibited these cytokines, highlighting the diverse regulatory roles of AhR ligands in macrophage-mediated immune responses.

## 4. The Role of AhR on Autophagy

AhR plays a key role in host defense mechanisms against both extracellular and intracellular bacterial infections. Moura-Alves et al. [61] identified AhR as a sensor for infections caused by *Pseudomonas aeruginosa* and *Mycobacterium tuberculosis*, highlighting its involvement in detecting and responding to bacterial pathogens. Certainly, AhR signaling has been found to contribute to the antibacterial response, particularly with a crucial involvement of macrophages and neutrophils during *P. aeruginosa* infection. These findings suggest that AhR likely plays a dual role during bacterial infections, promoting certain anti-bacterial activities while also reinforcing ‘disease tolerance’ mechanisms aimed at minimizing immunopathology in the host [62]. Antimicrobial peptides, such as human beta-defensin-3 (hBD-3) and cathelicidin LL37, exhibit not only antimicrobial activities but also immunomodulatory effects of cytokines/chemokines, as well as improving barrier function in epidermal keratinocytes [63,64]. AhR signaling takes part in both hBD-3-mediated mTOR- and MAPK-independent autophagy and improvement of the TJ barrier in keratinocytes [65].

A combination of indole-3-carbinol and genistein synergistically induces apoptosis in human colon cancer HT-29 cells by inhibiting the PI3K/Akt pathway and progression of autophagy [66]. In contrast, indole-3-carbinol-dependent signaling in plants induces specific autophagy in root cells [67] that can act as a general pathogen defense mechanism to repel insects.

## 5. The Role of AhR on Colitis

A deficiency in dietary AhR ligands worsens intestinal inflammation. Supplementing mice with Trp [68] or I3C [69] through oral gavage resulted in a mild improvement of DSS-induced colitis. Administration of FICZ, an AhR agonist, markedly alleviated the severity of colitis induced by TNBS, DSS, and T-cell transfer in mice. This improvement was associated with elevated IL-22 levels [70,71] and suppression of pro-inflammatory cytokines. In contrast, treatment with an AhR antagonist reduced IL-22 levels and exacerbated inflammation in a murine model of TNBS-induced colitis. Furthermore, blocking IL-22 with specific antibodies negated the anti-inflammatory effects of FICZ, suggesting that IL-22 plays a critical role in mediating FICZ’s therapeutic impact. Similarly, oral administration of another AhR agonist, β-naphthoflavone, was shown to decrease the severity of DSS-induced colitis while reducing pro-inflammatory cytokines such as tumor necrosis factor -α (TNF-α), IL-6, and IL-1β [28]. The toxic synthetic AhR ligand TCDD alleviated the severity of DSS-induced colitis by enhancing regulatory T cell (Treg) differentiation and suppressing Th17 cell induction, primarily through mechanisms involving epigenetic regulation [72]. I3C has been shown to alleviate colitis by promoting the induction of Tregs and suppressing proinflammatory Th17 cells, as well as reducing macrophage-driven cytokine production [69]. I3C specifically downregulates the expression of key proinflammatory cytokines, including IL-6, TNF-α, IL-1β, IL-23, and IFN-γ [73]. Both I3C and quercetin have demonstrated efficacy in mitigating chronic dextran sulfate sodium (DSS)-induced colitis in C57BL/6 mice through anti-inflammatory mechanisms mediated by the AhR [74]. Additionally, activation of AhR by I3C has been shown to reduce TNBS- or DSS-induced colitis and alleviate microbial dysbiosis resulting from colitis, and this effect occurs in an IL-22-dependent manner [41]. Treatment with I3C has demonstrated efficacy in treating patients with IBD, partially through the upregulation of IL-22, suggesting that targeting AhR could be a strategy to modulate the magnitude and duration of IL-22 signaling for the treatment of IBD patients, as well as I3C is being investigated as a natural therapeutic option for IBD. The microbiota from Card9(−/−) mice fails to metabolize Trp into metabolites that act as AhR ligands, and is more susceptible to colitis [71]. CARD9 promotes recovery from colitis by promoting AhR ligands and IL-22 production. Reduced production of AhR ligands is also observed in the microbiota from individuals with IBD, particularly in those with CARD9 risk alleles associated with IBD. Recently, we observed that an AhR inhibitor (CH-223191) counteracted the synergistic effects of postbiotic propionate (PP) and active VD3 on reducing the severity of *Salmonella* colitis in C57BL/6 mice. This was evidenced by a reduction in cecal inflammatory markers (e.g., IL-1β, TNF-α, and IL-6), enhancement of antimicrobial peptide (mBD-3) mRNA expression, and reduction in bacterial translocation to the liver and spleen, compared to single treatment [75]. These findings suggest AhR is involved in the synergistic effects of postbiotics PP and VD3 on antibacterial and anti-inflammatory responses in *Salmonella* colitis. This highlights the potential for biological treatment of *Salmonella* colitis.

## 6. The Role of AhR on Bacterial Infection

AhR ligand activation triggers essential immune responses, including IL-22 production, which plays a pivotal role in clearing intestinal pathogens. AhR-deficient (AhR^−/−^) mice exhibit heightened sensitivity to lipopolysaccharide (LPS)-induced septic shock, indicating that AhR helps safeguard the host against excessive immune reactions to Gram-negative bacteria [62,76,77,78]. Indeed, loss of AhR in mice results in higher susceptibility to *Citrobacter rodentium* [8,79,80], which is a mouse model widely used to mimic human infections caused by enteropathogenic and enterohemorrhagic Escherichia coli (*E. coli)* [81]. In response to LPS treatment, AhR-deficient (AhR^−/−^) mice exhibited elevated serum levels of IL-6, IL-1β, and TNF-α, alongside reduced IL-10 concentrations compared to wild-type (WT) mice. Similarly, macrophages from AhR^−/−^ mice secreted higher amounts of IL-6, IL-1β, and TNF-α following LPS stimulation compared to their WT counterparts [78]. In AhR^−/−^ mice or immune cells exposed to LPS, elevated levels of pro-inflammatory cytokines, including IL-1β, IL-18, IFN-γ, TNF-α, IL-12, and IL-6, have been observed. Additionally, increased expression of NLRP3, a key regulator of various pro-inflammatory cytokines, has been detected [77,82,83,84]. This heightened response is also observed in the context of Gram-positive intestinal pathogens. For example, *Listeria monocytogenes*, a facultative intracellular bacterium responsible for foodborne illness, is associated with significant morbidity and relatively high mortality rates. Mice lacking AhR exhibit increased susceptibility to *L. monocytogenes* [85], despite macrophages displaying heightened production of pro-inflammatory cytokines like IL-6 and TNF-α following infection [86]. The administration of the AhR ligand FICZ has been shown to confer protection against *L. monocytogenes* infection [45]. Recent research highlights the significant role of the AhR in the synergistic action of butyrate and vitamin D3 (VD3) to enhance host defenses against *Salmonella* infection. This combination improves intestinal barrier integrity by modulating tight-junction functions, thereby reducing bacterial invasiveness [7]. This effect was linked to increased expression of cecal cytokines and antimicrobial peptides, such as IL-17A, IL-22, mBD-3, and CRAMP, while reducing proteins like zonulin and claudin-2 to enhance tight junctions [7]. Furthermore, this combination therapy demonstrated potential beyond *Salmonella*, showing efficacy in treating gut-derived *Pseudomonas aeruginosa* sepsis in mice [87]. These findings suggest that the AhR pathway is central to the therapeutic benefits of combining postbiotics and VD3, presenting a promising alternative strategy for managing invasive bacterial infections.

## 7. AhR in Inflammatory Bowel Diseases

AhR expression is significantly reduced in patients with Crohn’s disease (CD), particularly in the inflamed mucosa, but remains unchanged in those with ulcerative colitis (UC) [88]. Moreover, patients with IBD have lower levels of endogenous AhR ligands compared to healthy individuals [10]. These findings suggest a potential link between AhR activity and IBD symptoms. Supporting this, murine models of dextran sulfate sodium (DSS)-induced colitis show more severe symptoms and elevated pro-inflammatory cytokine levels in AhR^−/−^ mice compared to WT controls [89]. Furthermore, The AhR ligands TCDD, Norisoboldine (NOR), and FICZ have been shown to alleviate colitis symptoms by activating AhR pathways [90,91,92]. TCDD exerts its effects by suppressing Th17 cell differentiation, leading to reduced expression of IL-17 and IFN-γ. In contrast, NOR mitigates colitis by enhancing regulatory T cell (Treg) differentiation and inhibiting the NLRP3 inflammasome [90,91]. FICZ provides protection against colitis by reducing the production of pro-inflammatory cytokines such as IL-17, IL-1β, IL-6, TNF-α, and IFN-γ, while promoting anti-inflammatory IL-22 production from Th17 cells [88,89,92]. IL-22 demonstrates rapid alleviation of local intestinal inflammation in a mouse model of Th2-mediated colitis resembling UC [93]. Reduced serum or plasma levels of Trp are observed in patients with IBD, particularly in those with Crohn’s disease (CD) [94,95]. This depletion of intestinal Trp catabolites, such as the AhR agonist indoleacetic acid (IAA), may contribute to the severity of IBD [71]. Curcumin, a natural compound, has been recommended as a complementary therapy for UC based on comprehensive meta-analyses. Moreover, a recent Japanese study demonstrated that Theracurmin^®^, a highly bioavailable curcumin derivative, improved clinical and endoscopic remission, healing of anal lesions, and levels of inflammatory markers in patients with active mild-to-moderate CD [96]. These findings suggest that AhR could serve as a crucial mediator in IBD, and dietary AhR ligands may represent promising therapeutic targets for the condition.

## 8. AhR in Celiac Disease

Celiac-disease-related intestinal inflammation is marked by accumulation of TNF-α producing ILCs in the gut mucosa, and depletion of ILCs preventing the poly I:C-driven intestinal damage [97]. AhR mRNA and protein expression is diminished in the intestinal mucosa of patients with active Celiac disease [98]. The AhR ligand FICZ demonstrated protective effects in mice against poly I:C-induced intestinal enteropathy. It notably reduced the levels of inflammatory cytokines and cytotoxic factors [98]. Gut-microbiota-dependent AhR ligand production and intestinal AhR pathway activation are decreased in celiac disease [99], leading to intestinal inflammation. These findings suggest a new therapeutic strategy for treating this disorder.

## 9. AhR in Colorectal Cancer (CRC)

Emerging evidence suggests that AhR and its ligands play crucial roles in intestinal tumorigenesis, though the exact role of AhR in carcinogenesis remains inconsistent [100]. Recent studies indicate that AhR signaling can have both pro- and anti-carcinogenic effects, potentially acting in a tissue-specific manner [100]. Natural AhR ligands derived from dietary Trp and glucoinolates have demonstrated efficacy in suppressing tumor formation in mouse models of CRC [101]. Furthermore, AhR functions as a tumor suppressor in liver carcinogenesis [102], and reduced AhR expression has been observed in human cecal cancer tissues and adjacent areas [101]. AhR may regulate intestinal tumorigenesis through its target genes [103] and also acts as a tumor suppressor in inflammation-associated intestinal neoplasia [104]. Given that chronic intestinal inflammation, as seen in inflammatory bowel diseases, increases the risk of CRC, AhR’s role in modulating inflammation may be key to its tumor-suppressing function [105]. Findings from tumor-forming mouse models indicated that the absence of AhR heightened colon carcinogenesis. Additionally, single-cell transcriptomics conducted on colonic intestinal crypts revealed that AhR deletion led to increased expression of Forkhead box protein M1 (FOXM1)-regulated genes across various colonic cell subtypes. Taken together, these findings strongly suggest a tumor-suppressor-like role of AhR in the colon. However, further translational studies are required to elucidate the role of AhR in various stages of colorectal cancer (CRC) progression and to guide AhR as a potential therapeutic target for CRC.

## 10. AhR in Liver Disease

A recent review has highlighted the role of the gut microbiota in the pathogenesis of chronic liver diseases, such as non-alcoholic fatty liver disease (NAFLD), non-alcoholic steatohepatitis (NASH), and liver cancer, within the context of the gut–liver axis [106]. Gut microbial dysbiosis is increasingly linked to the development of NAFLD and NASH, with studies showing that high-fat diet (HFD)-fed mice exhibit reduced levels of AhR ligands, including tryptamine and indole 3-acetate. These AhR ligands play an important role in suppressing the production of pro-inflammatory cytokines like TNF-α and IL-1β, which are implicated in liver inflammation and damage. Additionally, indole 3-acetate has been found to suppress the expression of AhR-regulated lipogenic enzymes, such as fatty acid synthase, as well as regulators of cholesterol metabolism like sterol regulatory element-binding protein-1c (SREBP-1c) in hepatocytes [107], which contributes to the prevention of NAFLD and NASH. This suggests that AhR signaling is crucial in modulating liver lipid metabolism and inflammatory responses, potentially offering a protective effect against these conditions. In the context of liver cancer, particularly hepatocellular carcinoma (HCC), the expression of AhR, along with indoleamine 2,3-dioxygenase-1 (IDO-1), kynurenine, and PD-L1, has been correlated with poor prognosis, indicating that the AhR pathway may also play a role in cancer progression and immune evasion [108]. This positions AhR as a key regulator in both metabolic liver diseases and liver cancer, with therapeutic potential in targeting AhR signaling for the treatment of these conditions.

Alteration of the intestinal microbiota has a significant impact on the immune system, particularly in disrupting the balance between regulatory Tregs and Th17 cells. This imbalance triggers an inflammatory response that leads to abnormal immune activation and promotes the development of autoimmune hepatitis (AIH). The AhR plays a critical role in regulating this balance. Activation of the AhR can induce the upregulation of CD39 (ectonucleoside triphosphate diphosphohydrolase 1), a molecule important for maintaining immune homeostasis. Low levels of CD39 are associated with an imbalance between Tregs and Th17 cells, contributing to immune dysregulation [109]. IL-17 further exacerbates this imbalance by impairing Treg cell function. Elevated IL-17 levels lead to the polarization of newly generated Tregs (ng Tregs) into a pro-inflammatory Treg phenotype with activated immune functions. This shift promotes immune cell infiltration, liver injury, and ultimately liver inflammation and fibrosis, all of which are hallmarks of AIH [110]. The inflammatory response driven by the Treg/Th17 imbalance, combined with AhR and IL-17 signaling, contributes to the progression of liver inflammation and fibrosis, underscoring the critical role of immune regulation in liver health.

## 11. AhR in Neurogenetic Diseases

The AhR plays a crucial role in the gut–brain axis, which is increasingly recognized as a significant factor in the development and pathology of various neurological disorders, including multiple sclerosis (MS) and its animal model, experimental autoimmune encephalomyelitis (EAE) [111]. Recent findings highlight that AhR is key in regulating immune responses, particularly through its control of the differentiation and stability of intestinal regulatory T cells (Tregs) [112]. Oral administration of ITE, an endogenous AhR agonist, has been shown to increase the ratio of myelin-reactive Tregs to effector T cells (Teffs), thereby suppressing the progression of EAE. These results suggest that AhR signaling contributes to anti-inflammatory effects not only within the gut but also in other tissues such as the central nervous system (CNS). This makes AhR a promising therapeutic target for immune-mediated diseases, as its modulation could potentially alleviate both intestinal and neurological inflammation. Modulation of the AhR by DIM has been shown to elicit neuroprotective effects against LPS-induced inflammation, safeguarding mouse neuronal cells from hypoxic damage in both in vitro and in vivo Parkinson’s disease models [113]. Additionally, the kynurenine (KYN)-AhR pathway has been identified as a crucial factor in mediating neuronal injury following experimental stroke, suggesting its potential as a therapeutic target for stroke treatment [114].

## 12. Conclusions

Accumulating evidence highlights the pivotal role of AhR in regulating inflammatory signaling, which significantly impacts the abundance and functionality of key immune cells involved in both innate and adaptive immunity, such as Th17 cells, Tregs, and dendritic cells (DCs) [115]. This deepening understanding paves the way for novel therapeutic strategies targeting various inflammatory conditions. As a central mediator, AhR orchestrates the complex interactions between intestinal immune cells, epithelial cells, and the gut microbiota, enhancing defense mechanisms, maintaining mucosal integrity, and mitigating harmful inflammation. This multifaceted function establishes AhR as a crucial regulator in host responses to infections, autoimmune disorders, and intestinal tumor development. Nutritional AhR ligands hold great promise as a therapeutic strategy for gut-mediated inflammation and diseases. By modulating immune responses, maintaining gut barrier integrity, and interacting with gut microbiota, these ligands have the potential to treat conditions like IBD, colorectal cancer, and other immune-related gut disorders, as summarized in Table 2. Ongoing research into specific dietary sources of AhR ligands, as well as optimizing their delivery and bioavailability, will be key in unlocking their full therapeutic potential. However, to fully realize the therapeutic potential of AhR-targeted interventions, further research is warranted to elucidate the long-term effects of these agents. Only then can we confidently consider their translation into clinical practice.

Harnessing microbiota-derived AhR ligands offers a promising avenue for novel therapeutic strategies. These approaches may include utilizing probiotics that produce AhR agonists or directly administering microbial-derived AhR agonists. Both strategies have shown effectiveness in alleviating experimental intestinal inflammation in colitis models, highlighting their potential for treating inflammatory conditions [116]. For instance, compounds like indole-3-carbinol have shown potential in mitigating the impact of infections such as *C. difficile* in murine models. Single-nucleotide polymorphisms that are near or distant from AhR binding sites have been shown to alter AhR ligand-dependent target gene expression that may contribute to inter-individual variation in disease prevalence, disease severity, and pharmacotherapeutic response [117]. Moreover, polymorphisms of the human AhR may also contribute to individual sensitivity to AhR ligand exposure [118], and several AhR single-nucleotide polymorphisms have been significantly associated with many diseases, including IBDs [119,120]. Moreover, recent advancements in antigen delivery via nanoparticles offer innovative opportunities to promote protective immune responses against both microbial pathogens and cancer. These findings collectively suggest a promising direction for the development of therapeutic interventions targeting the AhR pathway in inflammatory diseases [115].

**Table 2:** The role of nutritional aryl hydrocarbon receptor ligands in gut inflammation and diseases.

Diseases	AhR Ligands	Effects	Model	Mechanisms	Refs.
Colitis	Tryptophan [68] or I3C by oral gavage [69]	Amelioration of DSS-induced colitis	Mice	CARD9 promotes recovery from colitis by promoting AhR ligands and IL-22 production	[68,69]
	6-formylindolo[3,2-b] carbazole (FICZ) (Trp derivative)	Reduced the severity of Trinitrobenzene sulfonic acid (TNBS)-, DSS-, and T-cell-transfer-induced colitis	Mice	Increased production of IL-22 [70,71] and down-regulation of pro-inflammatory cytokines	[70,71]
	β-naphthoflavone (synthetic flavonoid)	Decrease the severity of DSS-induced colitis and the production of pro-inflammatory cytokines such as tumor necrosis factor (TNF)-α, IL-6, and IL-1β [28].	Mice and SW480 cells	Decrease the production of pro-inflammatory cytokines such as TNF-α, IL-6, and IL-1β [28]	[28]
	2,3,7,8-tetrachlorodibenzo-p-dioxin (TCDD) (cytotoxin)	The severity of DSS-induced colitis was attenuated	Mice	Increased differentiation of regulatory T cells (Tregs) and decreased induction of Th17 cells through epigenetic regulation [72]	[72]
Bacterial infection	Propionate (PP) and active vitamin D3 (VD3)	Reducing the severity of *Salmonella* colitis Reduction in bacterial translocation to the liver and spleen	Mice	A reduction in cecal inflammatory markers (e.g., IL-1β, TNF-α, and IL-6), enhancement of antimicrobial peptide (mBD-3) mRNA expression	[121]
	AhR-deficient (AhR^−/−^) mice	Detrimental immune responses toward Gram-negative bacteria	Mice	Hypersensitive immune responses to lipopolysaccharide (LPS)-induced septic shock	[62,76,77,78]
	Loss of AhR in mice	Higher susceptibility to *Citrobacter rodentium* [8,79,80].	Mice	Group 3 innate lymphoid cells inhibit T-cell-mediated intestinal inflammation through AhR signaling and regulation of microflora	[8,79,80]
	AhR^−/−^ mice	More highly sensitive to LPS-induced lethal shock	Mice	AhR forms a complex with Stat1 and nuclear factor-kappa B (NF-kappaB) in macrophages stimulated by LPS, which leads to inhibition of the promoter activity of IL-6	[86]
	Macrophages from AhR deficient (AhR^−/−^) mice	Secrete higher levels of IL-6, IL-1β, and TNF-α in macrophages stimulated by LPS	Mice	AhR negatively regulates IL-6 production via H1R signaling through the suppression of histamine production in macrophages following LPS stimulation	[78]
	AhR^−/−^ mice or immune cells	Increased concentrations of pro-inflammatory cytokines like interleukin 1 beta (IL-1β), IL-18, interferon gamma (IFN-γ), TNF-α, IL-12, and IL-6, along with NLR Family Pyrin Domain Containing 3 (NLRP3) in peritoneal macrophages upon exposure to LPS and suppresses Alum-induced peritonitis in vivo	Mice	AhR activation inhibits NLRP3 expression, caspase-1 activation, and subsequent IL-1beta secretion	[84]
	FICZ	Confer protection against *L. monocytogenes* infection [45]	Mice and RAW cells	Promotes macrophage survival through the induction of the antiapoptotic factor, the apoptosis inhibitor of macrophages Promotes ROS production for bacterial clearance	[45]
	Butyrate and VD3 [7].	Enhancing the host´s antibacterial defenses against *Salmonella* infection by preventing invasiveness	Mice	Regulating tight-junction functions	[7]
	Butyrate and VD3	Attenuate the severity of colitis and invasion of bacteria on gut-derived *Pseudomonas aeruginosa* sepsis [87]	Mice	Enhancement in the expression of defensive cytokines and antimicrobial peptides within the cecum, coupled with decreased levels of zonulin and claudin-2 proteins in the mucosal lining.	[87]
Inflammatory bowel disease	Trp	CARD9 promotes recovery from colitis, and Card9(−/−) mice are more susceptible to colitis. Reduced production of AhR ligands is also observed in the microbiota from individuals with IBD, particularly in those with CARD9 risk alleles associated with IBD	Mice and patients	CARD9 promotes recovery from colitis by promoting AhR ligands and IL-22 production. Host genes affect the composition and function of the gut microbiota, altering the production of microbial metabolites and intestinal inflammation.	[71]
	Theracurmin^®^, a highly bioavailable curcumin derivative	Improved clinical and endoscopic remission, healing of anal lesions, and levels of inflammatory markers in patients with active mild-to-moderate CD [96]	Patients	Reduced serum or plasma levels of Trp are observed in patients with IBD, particularly in those with Crohn´s disease (CD) [94,95]	[94,95,96]
Celiac disease	FICZ	Protective effects in mice against poly I:C-induced intestinal enteropathy [98]	Patients Mice	AhR mRNA and protein expression is diminished in the intestinal mucosa of patients with active Celiac disease [98]. Gut-microbiota-dependent AhR ligand production and intestinal AhR pathway activation are decreased in celiac disease [99] Reduced the levels of inflammatory cytokines and cytotoxic factors	[98,99]
Colorectal cancer	Trp and glucoinolates in the intestines	Have shown efficacy in suppressing tumor formation in mouse models of colorectal cancer (CRC) [101]	Mice	AhR may regulate intestinal tumorigenesis through its target genes (e.g., CYP1A1) [103] Also acts as tumor suppressor in inflammation-associated intestinal neoplasia[104] AhR deletion led to increased expression of Forkhead box protein M1 (FOXM1)-regulated genes across various colonic cell subtypes	[101,103,104]
Autoimmune hepatitis	TCDD [122]	Strategies aimed at targeting factors that disrupt the AhR canonical pathway or directly enhancing CD39 expression and activity [39,123]	Mice	Dysregulation in the expression and control of CD39 is observed in both Tregs and Th17 cells derived from individuals with AIH [124]	[122,124]
Autoimmune encephalomyelitis	TCDD FICZ	Suppressed experimental autoimmune encephalomyelitis Increased the severity of experimental autoimmune encephalomyelitis in mice	Mice	Induced functional T(reg) cells that suppressed experimental autoimmune encephalomyelitis Interfered with T(reg) cell development, boosted T(H)17 cell differentiation	[39]
	3. AhR activation during induction of experimental autoimmune encephalomyelitis	Accelerated onset and increased pathology in wild-type mice, but not AhR-deficient mice	Mice	3. Activation of AhR by a high-affinity ligand during T_H_17 cell development markedly increases the proportion of T_H_17 T cells and their production of cytokines	[123]

## Figures and Tables

**Table 1:** Summary of the nutritional classification of aryl hydrocarbon receptor (AhR) ligands.

Category	Source/Examples	Role/Implications
Endogenous ligands		
- Tryptophan derivatives	Indole-3-acetic acid (I3A)	Modulate immune responses, gut health, and maintain immune cell balance in the gut to obtain immune tolerance
	Kynurenic acid (KA)	
	Kynurenine	
	2-oxindole	
	Tryptamine (TA)	
	3-methyl indole	
	Indole-3-aldehyde (IAld)	
- Microbiota-derived compounds	Indole derivatives (Indole-3-propionic acid)	Impact gut health and immune function, derived from the microbial fermentation of fibers
	Short-chain fatty acids (SCFAs)	
Exogenous ligands (dietary)		
- Polyphenols	Quercetin, resveratrol (found in fruits, vegetables, teas)	Anti-inflammatory and anti-cancer properties, regulate immune responses
- Carotenoids	Beta-carotene (found in colorful fruits and vegetables)	Possible activation of AhR; research ongoing on its impact on immune regulation
- Cruciferous vegetable compounds	Indole-3-carbinol (broccoli, cauliflower, cabbage)	Known AhR activators, potential anti-cancer effects
	Indole-3-acetonitrile (IAN)	
Environmental ligands		
- Pollutants	Dioxins, polycyclic aromatic hydrocarbons (PAHs)	Negative health effects including toxicity and inflammation
Role in nutritional immunology	AhR activated by dietary ligands affects immune balance	Supports immune modulation and regulation of gut health
Metabolic regulation	Activation by dietary ligands affects energy metabolism	Implications in obesity, type 2 diabetes, and metabolic disorders
Therapeutic potential	Foods high in AhR ligands (e.g., cruciferous vegetables, polyphenols)	Potential for managing inflammation, cancer prevention, and metabolic diseases
